# O.6.1-9 Motor coordination as a strategy to increase school achievement in schoolchildren

**DOI:** 10.1093/eurpub/ckad133.268

**Published:** 2023-09-11

**Authors:** Douglas Vieira, Mabliny Thuany, Thayse Natacha Gomes

**Affiliations:** Post Graduation Program of Physical Education, Federal University of Sergipe; Faculty of Sports, University of Porto; Post Graduation Program of Physical Education, Federal University of Sergipe; Department of Physical Education and Sport Sciences, Physical Activity for Health Research Cluster, Health Research Institute, University of Limerick; douglasavieira9889@gmail.com

## Abstract

**Purpose:**

Our purpose was to identify the role of demographic, attention level, motor coordination, and physical activity to predict school achievement in Brazilian schoolchildren.

**Methods:**

This is a cross-sectional study, composed of 106 Brazilian schoolchildren of both sexes (56 boys), aged between 7-12 years. Sociodemographic characteristics (age, sex, socioeconomic status - SES), motor coordination (KTK test), physical activity (WEB CAAF questionnaire), attention level, and school performance (measured by school performance test and categorized based on the median in “insufficient” or “good”) were obtained. A hierarchical logistic regression analysis was estimated to verify factors associated with academic achievement (“insufficient achievement” and “good achievement”), through three models increased in complexity (model 1: age, sex, SES; Model 2: Model 1 + attention variables; Model 3: Model 2 + motor coordination and physical activity). Effect size estimates were computed using R^2^. The SPSS software was used, with a confidence interval fixed at 95%. The research was approved by the research ethics committee of the Federal University of Sergipe – Brazil (n° 5.155.350).

**Results:**

About 51% of the children presented insufficient school achievement. Based on the three logistic regression models, the variables associated with school achievement included age (OR = 2.32; 95%IC = 1.23 – 4.37), and attention level (OR = 1.07; 95%IC = 1.01 – 1.14). Results from the logistic regression showed that the model which better explained school achievement variance was the model 3, where all the variables were included (R2=66%). Higher motor coordination increased the odds to present good school achievement (OR = 10.42, 95%IC = 1.04 – 104.46), but no significant association was shown for physical activity.

**Conclusions:**

Interventions to improve school achievement among Brazilian schoolchildren should consider the use of motor coordination, including activities that require focus, especially in young children.

